# Cellular Effects of Rhynchophylline and Relevance to Sleep Regulation

**DOI:** 10.3390/clockssleep3020020

**Published:** 2021-06-09

**Authors:** Maria Neus Ballester Roig, Tanya Leduc, Cassandra C. Areal, Valérie Mongrain

**Affiliations:** 1 Center for Advanced Research in Sleep Medicine, Recherche CIUSSS-NIM, Montréal, QC H4J 1C5, Canada; mneusballester@gmail.com (M.N.B.R.); tanyaleduc@hotmail.com (T.L.); cassandra.areal@gmail.com (C.C.A.); 2 Department of Neuroscience, Université de Montréal, Montréal, QC H3T 1J4, Canada; 3 Department of Medicine, Université de Montréal, Montréal, QC H3T 1J4, Canada

**Keywords:** *Uncaria rhynchophylla*, intracellular signaling pathways, neurotransmitter receptors, non-rapid eye movement sleep, rapid eye movement sleep, electroencephalographic activity

## Abstract

*Uncaria rhynchophylla* is a plant highly used in the traditional Chinese and Japanese medicines. It has numerous health benefits, which are often attributed to its alkaloid components. Recent studies in humans show that drugs containing *Uncaria* ameliorate sleep quality and increase sleep time, both in physiological and pathological conditions. Rhynchophylline (Rhy) is one of the principal alkaloids in *Uncaria* species. Although treatment with Rhy alone has not been tested in humans, observations in rodents show that Rhy increases sleep time. However, the mechanisms by which Rhy could modulate sleep have not been comprehensively described. In this review, we are highlighting cellular pathways that are shown to be targeted by Rhy and which are also known for their implications in the regulation of wakefulness and sleep. We conclude that Rhy can impact sleep through mechanisms involving ion channels, *N*-methyl-d-aspartate (NMDA) receptors, tyrosine kinase receptors, extracellular signal-regulated kinases (ERK)/mitogen-activated protein kinases (MAPK), phosphoinositide 3-kinase (PI3K)/RAC serine/threonine-protein kinase (AKT), and nuclear factor-kappa B (NF-κB) pathways. In modulating multiple cellular responses, Rhy impacts neuronal communication in a way that could have substantial effects on sleep phenotypes. Thus, understanding the mechanisms of action of Rhy will have implications for sleep pharmacology.

## 1. Introduction

Plant compounds have been substantially explored to treat human illnesses, especially in the traditional Chinese medicine. This includes their utilization to ameliorate sleep or induce sedation [1,2]. However, given that the use of such compounds began early in the human history, the knowledge of their beneficial effects on health is rarely accompanied by studies providing the details of the underlying mechanisms.

*Uncaria rhynchophylla* has been used in Asia as a component of numerous Chinese and Japanese treatments such as Gou-teng (or Chotoko; name given to *Uncaria* medicinal herbs), and Yi-gan-san (a blend of seven herbs also known as Yokukansan [YKS]). It has been reported to alleviate hypertension, arrhythmia, convulsions, dizziness, pain, sleep disturbances, and cognitive impairments [3,4,5,6,7,8]. Alkaloids account for 0.2% of the composition of *U. rhynchophylla* (in hook, stem, and leaves) and were proposed to underlie the majority of health benefits resulting from the use of *Uncaria* [4,9]. Rhynchophylline (Rhy) is one of the most abundant of these alkaloids and seems to associate with nearly the same benefits as those obtained with *U. rhynchophylla* in nonhuman mammals [3,4,10].

### 1.1. Rhynchophylline Pharmacology

**Figure 1:**
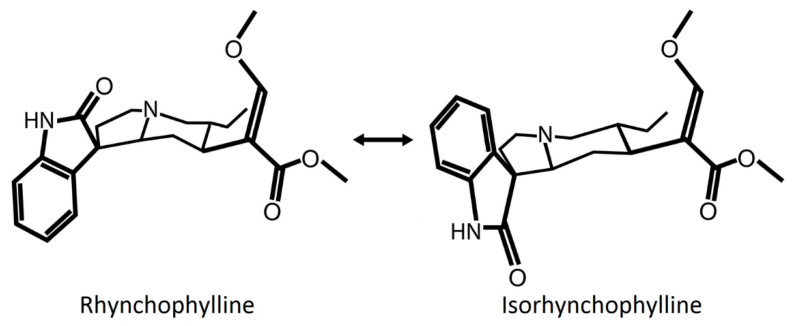
Representation of the chemical structure of Rhynchophylline (Rhy) and Isorhynchophylline (Isorhy). The position of the oxindole structure (N-C=O in the second ring) of the alkaloids Rhy and Isorhy is different. Both molecules are diastereoisomers, interconvertible with each other depending on pH and temperature. Temperature is suggested to induce a break and reclosure of the third ring that results in a twisted conformation [14].

Rhy is a tetracyclic oxindole alkaloid that represents about 10–30% of *Uncaria* alkaloids [9,11,12]. Rhy is interconvertible with its isomer isorhynchophylline (Isorhy), which accounts for another 30–50% of the alkaloid fraction [9,11,12] (Figure 1). Their rate of interconversion depends on pH and temperature [13,14]. Both forms are absorbed quickly by the intestine but, when provided intravenously or orally, Rhy seems more available than Isorhy in the plasma, likely because the latter is more unstable and metabolized faster by the liver and intestine [13]. Rhy easily crosses the blood–brain barrier, as it is highly detectable in the rat brain from 15 min to 6 h after oral administration [15]. Another study has shown that an *in vitro* blood–brain barrier model was more permeable to Isorhy than Rhy [16]. Therefore, even if Rhy could be more prevalent than Isorhy in the body, the administration of Rhy may trigger the presence of Isorhy, which effect should be considered.

Rhy (like Isorhy) has been proposed to mainly act on the cardiovascular system and central nervous system (CNS) [3,10]. Although there is no clinical trial investigating the effects of Rhy alone, animal research suggests that Rhy has beneficial properties such as anti-inflammatory, antihypertensive, anti-arrhythmic, anticonvulsant and neuroprotective effects [3,10]. Moreover, it seems to reduce memory impairments, mood dysregulation, and addictive behaviors in rodents [17,18,19,20,21]. Interestingly, one study [22] and recent unpublished data from our group point to an effect of Rhy on sleep in rodents, which is in line with the beneficial effects of Chotoko and YKS on human sleep time and quality (see details in Section 1.3).

### 1.2. Sleep and Its Regulation 

In mammals and other species, sleep is an essential behavior during which the organism isolates from environmental stimuli. Although the precise roles of sleep remain elusive, it could serve recovery from sustained activity (and associated oxidative stress) occurring during wakefulness in mammals and insects [23,24]. Moreover, sleep is beneficial for immune function, memory consolidation, and mood [25,26,27,28]. Mammalian sleep studies usually identify three main vigilance states: wakefulness, non-rapid eye movement (NREM) sleep (analogous to slow wave sleep in rodents), and rapid eye movement (REM) sleep (or paradoxical sleep) [29]. Wakefulness is characterized by a predominance of high frequency electroencephalographic (EEG) activity, NREM sleep by predominant low-frequency and high-amplitude EEG activity, and REM sleep by theta (4–9 Hz) EEG activity [29,30,31,32,33]. Delta activity (1–4 Hz) and slow oscillations (<1 Hz) during NREM sleep originate from synchronized up and down states of neuronal firing in cortical and thalamocortical networks [34,35]. Delta activity (or slow wave activity: 0.5–4.5 Hz) was proposed to reflect a sleep homeostatic/recovery process [31,32,36,37,38], which relationship was recently shown to differ between slower and faster delta [32].

The transitions between vigilance states are operated by the activation/inhibition of specific brain circuits [39,40]. During wakefulness, wake-promoting brain regions contribute to sustained neuronal activity and/or inhibit sleep promoting centers. Amongst the major wake-promoting centers are Hypocretin/Orexin neurons in the lateral hypothalamus, neurons in the basal forebrain (BF), and neurons in several nuclei of the reticular formation (laterodorsal tegmentum [LDT], pedunculopontine tegmentum [PPT], raphe nucleus [RN], locus cœruleus [LC]) [39,41,42,43,44,45,46]. Sleep promoting neurons are found in the hypothalamus, with the ventrolateral preoptic area having a particular relevance [47]. During REM sleep, neurons from several nuclei of the reticular formation, including the LDT and PPT, allow cortical activation while behavioral sleep is maintained [48,49]. The knowledge of sleep neurobiology is important to refine pharmacological approaches for sleep disturbances.

### 1.3. Rhynchophylline and Sleep

Drugs containing *Uncaria* appear to ameliorate sleep in different ways. For instance, YKS was shown to improve sleep disturbances (sleep time, quality, and sleep-related limb movements) in adults suffering from REM sleep behavior disorder or dementia [6,50,51,52]. It was also reported to improve sleep quality in patients with insomnia [7] and children with nocturnal enuresis [53]. Other drugs containing *Uncaria* (although in smaller proportion) were also shown to increase total sleep time in healthy subjects and sleep quality in patients with Parkinson’s disease or perimenopausal sleep disorder [54,55,56]. Fundamental research also suggests that *Uncaria* benefits sleep in rodents. Indeed, the administration of both YKS and a drug containing YKS was found to increase sleep time in socially isolated mice while having no impact in group-housed mice [57,58]. YKS was also shown to increase NREM sleep (and to decrease wake time) in a rat model of dementia [59], and Chotoko was reported to enhance the hypnotic-induced sleep time in mice [60]. Interestingly, Yoo and collaborators showed that Rhy increases sleep time in wild-type rats and mice [22]. This is in line with our recent observation of a longer time spent asleep after Rhy administration in mice, especially during the active (dark) period (Ballester Roig et al., in preparation). Moreover, Rhy, Isorhy or *Uncaria* were all shown to reduce spontaneous locomotor activity in mice [61,62,63].

**Figure 2:**
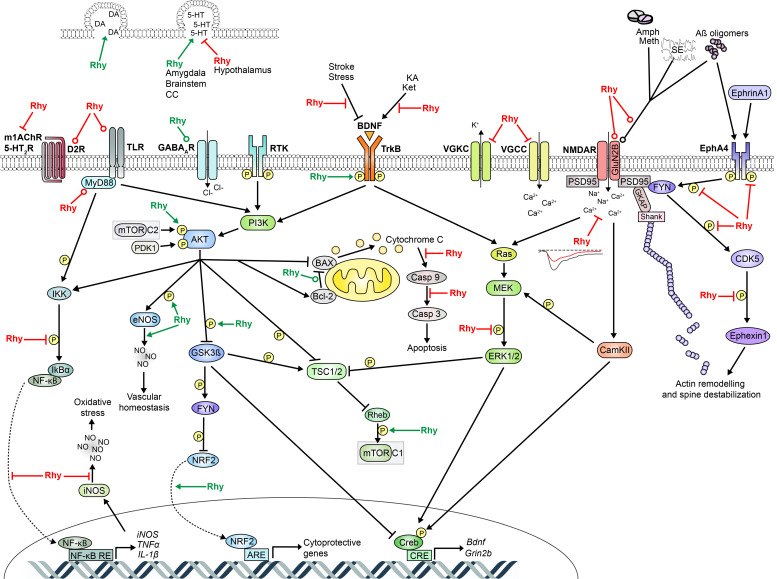
Schematic representation of cellular pathways targeted by Rhy and relevant to sleep regulation. Red flat-head lines: Rhy inhibition; Green arrows: Rhy induction; Red round-head lines: Rhy-dependent decrease in expression level; Green round-head lines: Rhy-dependent increase in expression level. Additional interactions between these cellular pathways are not represented but could also be relevant to sleep molecular physiology. For instance, L-VGCC can activate ERK/MAPK [144] and are suggested to induce CaMKII, NR2B phosphorylation, and CREB activation [106,145]. NMDARs may also activate the PI3K/AKT pathway [143]. In addition, NF-κB and ERK/MAPK pathways were shown to interact with each other [82,142]. 5-HT: 5-hydroxytryptamine or serotonin; 5-HT2R: serotonin receptor 2; Aβ: amyloid β; Amph: amphetamine; AKT: RAC serine/threonine-protein kinase; ARE: antioxidant response element; BAX: Bcl-2 associated X protein; BDNF: brain-derived neurotrophic factor; CamKII: Ca^2+^/calmodulin-dependent protein kinase; Casp 3: caspase 3; Casp 9: caspase 9; CC: cerebral cortex; CDK5: cyclin dependent kinase 5; CRE: cAMP response element; CREB: cAMP response element-binding protein; D2R: dopamine D2 receptor; DA: dopamine; eNOS: endothelial nitric oxide synthase; EphA4: Eph receptor A4; ERK1/2: extracellular signal-regulated kinases 1 and 2; FYN: tyrosine-protein kinase Fyn; GABA_A_R: gamma-aminobutyric acid type A receptor; GKAP: guanylate kinase-associated protein; GluN2B: NMDAR subunit 2B; *Grin2b*: glutamate ionotropic receptor NMDA type subunit 2B gene; GSK3β: glycogen synthase kinase-3 β; IκBa: NF-kappa-B inhibitor alpha; IKK: IκB kinase; IL: interleukin; iNOS: inducible nitric oxide synthase; KA: kainic acid; Ket: ketamine; m1AchR: m1-type muscarinic acetylcholine receptor; MEF2D: myocyte enhancer factor 2D; MEK: mitogen-activated protein kinase kinase; Meth: methamphetamine; mTOR: mechanistic target of rapamycin; mTORC1: mTOR complex 1; mTORC2: mTOR complex 2; MyD88: myeloid differentiation primary response protein; NF-κB: nuclear factor-kappa B; NMDAR: N-methyl-D-aspartate receptor; NO: nitric oxide; NRF2: nuclear factor E2 related factor 2; PDK1: phosphoinositide-dependent protein kinase-1; PI3K: phosphoinositide 3-kinase; PSD95: postsynaptic density protein 95; RE: response element; Rheb: GTP-binding protein Rheb; Rhy: rhynchophylline; SE: status epilepticus; Shank: SH3 and multiple ankyrin repeat domains protein; TLR: toll-like receptors; TNFα: tumor necrosis factor α; TrkB: tropomyosin or tyrosine receptor kinase B; TSC1/2: tuberous sclerosis complex 1/2; VGCC: voltage-gated calcium channel; VGKC: voltage-gated potassium channel.

Very few of these studies have investigated the cellular pathways underlying modifications of sleep. Three of them suggested that the increased sleep time in mice is linked to gamma-aminobutyric acid (GABA) neurotransmission because these effects were blocked by GABA receptor antagonists and since increased levels of GABA_A_ receptor subunits were found in hypothalamic neurons following Rhy-containing drug administration (see also Section 2.8) [22,57,58]. Another study in rats with cerebral ischemia has linked the effects of YKS on sleep to a change in the mRNA level of prostaglandin receptors in the prefrontal cortex (PFC) and hypothalamus [59]. However, it appears that multiple cellular pathways impacted by Rhy may drive modifications in sleep. Therefore, this review is assembling findings on potential targets and cellular pathways affected by Rhy that are likely to impact the regulation of sleep. The literature demonstrates that Rhy could affect the activity of ion channels, *N*-methyl-d-aspartate (NMDA) receptors, receptor tyrosine kinases (RTK), extracellular signal-regulated kinases (ERK)/mitogen-activated protein kinases (MAPK), phosphoinositide 3-kinase (PI3K)/RAC serine/threonine-protein kinase (AKT), and nuclear factor-kappa B (NF-κB). A detailed overview of the effects of Rhy, including different types and durations of administration, is presented in Table 1. In addition, Table 2 lists the literature reporting effects of Rhy on specific sleep-relevant targets/pathways, and Figure 2 depicts a global scheme of the sleep-relevant pathways affected by Rhy and their interrelationships.

## 2. Rhy Targets and Links to Sleep Regulation

### 2.1. Ion Channels

#### 2.1.1. Voltage-Gated Calcium Channels

Rhy was first described as a calcium channel blocker in arteries, heart and neuronal cultures from the rat, rabbit, guinea pig, and human [10]. Some studies suggest an inhibitory effect specifically on L-type voltage-gated calcium channels (L-VGCCs; Cav1 family of calcium channels), which are high-voltage activated channels present notably in neurons, retinal photoreceptors, vascular smooth muscle cells, and cardiomyocytes [102]. For example, acute *in vitro* incubation of rat cortical neurons, rat ventricular myocytes, and rat and human arteries with Rhy was shown to inhibit Ca^2+^ influx through L-VGCCs [66,67,69,103] (Table 1). In vessels, this Rhy-dependent inhibition of VGCCs and the inhibition of intracellular Ca^2+^ release were found to block the contractile response and induce vasodilation [69,103,104]. In cortical neurons, it was suggested that Rhy blocks L-VGCCs by decreasing the channel opening time and increasing its closing time under hypoxic conditions [66]. In neurons, L-VGCCs are mainly postsynaptic and contribute to Ca^2+^ influx, Ca^2+^ intracellular signaling, neuronal firing, and synaptic plasticity [105,106,107,108]. These roles affect neuronal responsiveness and synchronization, which is relevant to sleep regulation.

L-VGCCs were shown to modulate the synchronization of cortical and hippocampal neuronal oscillations, including in theta frequencies *in vitro* [109,110], and to affect the excitation/inhibition ratio in cortical slices [111]. In fact, Ca^2+^ signaling and ion channels including VGCCs are also proposed to be involved in the generation of the up and down states composing the slow oscillations characteristic of the NREM sleep EEG [112,113]. Cav1.2 channels represent more than 80% of L-VGCCs in the mouse brain [114]. Mice heterozygous for *Cacna1c* (gene encoding a Cav1.2 subunit) have less REM sleep during recovery after sleep deprivation (SD), as well as decreased beta and gamma activity (20–64 Hz) during wakefulness and REM sleep [115]. In addition, *Cacna1c* genetic variants, which have also been linked to psychiatric disorders, are associated with longer sleep latency in infants [116]. Therefore, although the effect of Rhy on neuronal L-VGCCs seems to have only been studied *in vitro*, Rhy may impact sleep stages and EEG activity through the blockage of L-VGCC-mediated currents. Moreover, Cav1.2 mRNA is expressed rhythmically in the mouse suprachiasmatic nucleus (SCN), and Cav1.2 KO mice have altered circadian adjustments to light [117]. This suggests that the effect of Rhy on VGCCs may also impact the circadian regulation of wakefulness and sleep.

#### 2.1.2. Potassium Channels

Other ion channels targeted by Rhy which have important roles in CNS functions are voltage-gated potassium channels (VGKC). VGKC, by allowing K^+^ efflux, regulate neuronal repolarization and the timing of neuronal excitability [118]. Rhy was shown to speed up the inactivation of VGKC in N2A neuroblastoma cells [64] (Table 1). This study has also reported a specific effect on VGKC containing the Kv1.2 subunit expressed in HEK293 cells, in which Rhy accelerated Kv1.2 channels activation and inactivation times [64]. Noteworthy, the Kv1.2 subunit is highly expressed in the thalamocortical system [119,120], and potassium channels Kv1.2, Kv3.1 and Kv3.2 have been shown to regulate sleep [121,122,123,124]. In particular, Kv1.2 knockout (KO) mice spend less time in NREM sleep and more time in wakefulness [122], and Kv1.2 inhibition was reported to decrease NREM sleep and alter the NREM sleep EEG [124]. In *Drosophila*, mutation of VGKC subunits that are close to the mammalian Kv1.2 channels was also shown to induce a decrease in sleep time [24,125]. These findings suggest that the effect of Rhy on VGKCs may contribute to alterations in sleep features as well. Of note, Rhy also affects calcium-activated potassium channels in the vascular system [10]. This has not been investigated in the CNS but might be of relevance considering that these channels can impact sleep duration [126]. Interestingly, both VGKCs and calcium-activated potassium channels are also suggested to be involved in the generation of up and down states of NREM sleep oscillations [112,113].

### 2.2. NMDA Receptors

Among the most studied targets of Rhy are glutamate NMDA receptors (NMDARs), which are crucial for neurotransmission and brain plasticity [127]. Rhy was described as a non-competitive NMDAR antagonist due to its blocking effect on NMDAR current in xenopus oocytes [68]. In entorhinal cortex slices of epileptic rats, Rhy was found to cause an immediate attenuation of the potentiated NMDAR-mediated currents, which was associated to a decrease of seizures *in vivo* [19]. Moreover, Rhy was often shown to decrease the expression of the NMDAR subunit GluN2B, which is predominant in extrasynaptic NMDARs, responds to high spreads of glutamate such as in excitotoxic conditions, and activates apoptotic pathways [128,129]. In rodents, conditions such as pilocarpine-induced status epilepticus, injections of amyloid-beta (Aβ), and administration of amphetamine (amph) or methamphetamine (meth), are increasing GluN2B protein levels, effects that were diminished by Rhy in the medial PFC, entorhinal cortex, and hippocampal CA1 region [19,20,83,93] (Table 1 and Table 2). This modulation of GluN2B by Rhy could depend on an effect at the gene expression level because Rhy was shown to reduce *Grin2b* mRNA levels in rat hippocampal neurons and also after an amph-induced increase in PFC and CA1 [20,72]. Additionally, the effects of Rhy on NMDAR and GluN2B have been linked to a decrease in the frequency of discharge or population spike amplitude in brain regions including the entorhinal cortex and dentate gyrus (DG) [19,80,83]. Moreover, the Rhy-driven decreases in GluN2B are often observed in parallel with improvements in cognitive functions in rodents, such as spatial memory or drug-conditioned place preference (CPP) [20,83,93]. Similar findings were made in the zebrafish, in which Rhy was found to reduce the meth-induced increase in GluN2B protein level and CPP [88]. In contrast to the aforementioned studies, Rhy was shown to increase GluN2B protein in human mesenchymal cells [78]. Despite the fact that these last findings were from relatively long bath incubations of Rhy (72 h), they are difficult to reconcile with most of the effects reported *in vivo* in rodents. Also, it is important to keep in mind that only one study has reported an effect of Rhy on NMDARs in baseline conditions, and this was *in vitro*, which may raise the question whether Rhy can modulate NMDARs under baseline conditions *in vivo*. Nonetheless, the literature adds up in favor of an effect of Rhy on NMDAR function.

With regard to sleep, glutamatergic signaling and NMDARs have been implicated both in arousal- and sleep-promoting pathways, with very distinct implications depending on the brain region [39]. On the one hand, NMDA or glutamate injected in the rat BF or tuberomammillary nucleus was shown to increase time spent awake [130,131], and injection of glutamate in the PPT induces neocortical desynchronization, wakefulness and REM sleep in the rat and cat [132,133]. Similarly, intraperitoneal (i.p.) injection of the MK-801 NMDAR antagonist was found to cause a delayed increase in NREM sleep time in rats [134,135]. Also, Alzheimer’s disease patients treated with a non-competitive antagonist of NMDARs showed an increase in total sleep time (mainly NREM sleep), and reduced sleep fragmentation [136]. On the other hand, glutamate injection in the rat medial preoptic area (mPOA) or medial septum was shown to promote NREM sleep [137,138], and MK-801 was reported to decrease both NREM and REM sleep in mice [126]. Other data in rats have shown that peripheral administration of NMDAR antagonists induces cortical gamma activity (30–50 Hz) in all vigilance states, while a specific blockade of GluN2B increases it solely in REM sleep [139]. The discrepancies between some of these studies could be explained by differences in the time of administration, time of recording, and/or species. Nonetheless, all support a role for NMDAR-mediated neurotransmission in sleep regulation. Therefore, the ‘generally antagonistic’ effect of Rhy on NMDARs should modulate cortical activity and show vigilance state-specific effects on wake/sleep architecture and EEG activity. Moreover, downstream effectors of NMDARs, including components of the ERK/MAPK and PI3K/AKT pathways, also seem to be altered by Rhy and involved in sleep regulation [82,94,140,141,142,143] (Figure 2, and Section 2.5 and Section 2.6). These interrelationships may reinforce the association between Rhy and NMDARs but could also imply that Rhy affects these pathways in a NMDAR-independent manner.

### 2.3. EphA4 and Downstream Pathways

Ephrins and their Eph RTKs are cell adhesion molecules widely expressed in neurons, glia, lymphocytes, epithelial cells, fibroblasts, myocytes, and bone cells [146,147,148,149]. In the CNS, they are crucial for axon guidance and plasticity [150]. In particular, Eph receptor A4 (EphA4) has roles in the regulation of α-amino-3-hydroxy-5-methyl-4 -isoxazolepropionic acid (AMPA) receptors, glial glutamate transport, and spine morphology [150,151,152]. In 2014, Fu and collaborators proposed that Rhy inhibits EphA4 activation by direct high-affinity interaction with its extracellular domain [18]. In this study, it was shown that Rhy inhibited both the EphrinA1-induced and Aβ-induced phosphorylation of EphA4 in rat hippocampal neurons, and that oral administration of Rhy inhibited the elevated phosphorylation of EphA4 in the hippocampus of mice mutant for the amyloid precursor protein (APP) and presenilin 1 (PS1) [18]. These observations were associated with a restorative effect of Rhy on long-term potentiation and spine number. A subsequent study also showed that one Rhy i.p. injection reduces p-EphA4 in mice susceptible to stress, specifically in the PFC, hippocampal CA3, and DG, which correlated with an improvement of depressive-like behaviors and spine number [17]. In these same stress-susceptible mice, the phosphorylation of the tyrosine-protein kinase Fyn, cyclin dependent kinase 5 (Cdk5) and ephexin1 was increased, and Rhy attenuated these increments [17]. This could originate from an effect of Rhy directly on EphA4 because the Cdk5/ephexin1 pathway is downstream of EphA4 phosphorylation and linked to actin remodeling and spine destabilization [153] (Figure 2).

Research from our group supports a role for EphA4 in the regulation of sleep [154,155]. Indeed, we found that *EphA4* KO mice spend less time in REM sleep and have longer bouts of wakefulness and NREM sleep during the light phase in comparison to wild-type littermates [154]. Also, *EphA4* KO mice manifested a blunted 24-h rhythm of NREM sleep sigma (10–13 Hz) activity [154]. In addition, *EphA4* KO mice showed a shorter duration of slow waves (0.5–4 Hz) during NREM sleep [155]. These observations suggest that Rhy might modulate sleep through EphA4-dependent pathways, which may alter sleep variables such as REM sleep amount or EEG properties in the sigma or delta frequency ranges. In parallel, *EphA4* was shown to be expressed in the mouse and rat SCN, and *EphA4* KO mice to have altered circadian responses to light [154,156]. This suggests an implication of EphA4 in the circadian timing system and, as a consequence, a potential effect of Rhy on circadian physiology.

### 2.4. BDNF/TrkB Signaling

Brain-derived neurotrophic factor (BDNF) is upregulated by neuronal activity and involved in cell survival and neuroplasticity [157,158,159,160,161]. It generally acts on p75 neurotrophin receptor (p75NTR) and tropomyosin or tyrosine receptor kinase B (TrkB) [162], and TrkB can activate other signaling pathways including PI3K and ERK/MAPK [160,163,164,165,166]. In a rat model of epilepsy, kainic acid was found to increase BDNF protein in the cerebral cortex and hippocampus, which was attenuated by Rhy or *Uncaria* [85]. Similarly, ketamine-addicted rats were shown to have an increased expression of BDNF in the hippocampus, which was diminished by Rhy [21,92]. Rhy was also observed to reduce the levels of extracellular and intracellular BDNF in human bone marrow mesenchymal cells [78]. In contrast, Rhy appears to restore BDNF level when it is decreased in pathological conditions instead of increased, such as in the cortex or hippocampus of a rat stroke model [94] or of chronic/social-defeat stressed mice [17,99]. TrkB phosphorylation was also found to be increased by Rhy in the PFC, hippocampal CA3 and DG regions of stressed mice, and in the striatum of a rat model of Tourette syndrome [17,96]. Therefore, Rhy may downregulate the BDNF pathway under some conditions of neuronal activation such as epilepsy or after ketamine administration, while it may upregulate it in specific pathological conditions such as stroke, stress or Tourette syndrome (Table 1). This could also suggest that Rhy effects on BDNF depend on distinct upstream pathways.

Both BDNF and TrkB signaling have been linked to sleep regulation [167,168,169]. Firstly, BDNF has long been considered a sleep-promoting substance. For example, intracerebroventricular injection of BDNF was found to induce NREM sleep in rats and NREM and REM sleep in rabbits [170]. Studies in humans also report that lower levels of BDNF associate with shorter sleep duration or with decreased amount of deep NREM and REM sleep [171,172]. Interestingly, TrkB KO mice have more REM sleep, reduced REM sleep latency, and shorter bouts of wake and NREM sleep [173]. Secondly, the BDNF/TrkB pathway was found to impact the sleep EEG. Indeed, intracerebroventricular injection of BDNF was shown to reduce NREM sleep slow wave activity (SWA) in rabbits [170], whereas BDNF injection in the rat cortex during wakefulness was shown to increase SWA in the following NREM sleep period, and cortical injection of a BDNF antibody or a TrkB inhibitor to reduce NREM sleep SWA [174]. Moreover, the Val66Met *BDNF* polymorphism in humans has been linked to decreased NREM sleep delta and theta activity, and REM sleep theta, sigma and alpha activity [175,176]. Carriers of this polymorphism also lost the positive correlation between sleep consolidation and declarative memory [177]. Thirdly, the phosphorylation of BDNF and TrkB responds to SD. Acute SD was shown to enhance BDNF levels and p-TrkB in the rat BF [178], and REM sleep deprivation (RSD) to increase BDNF in the PPT and subcœruleus nucleus, as well as in the ventromedial medulla of the spinal cord in a rat pain model [179,180,181]. SD was also found to increase BDNF levels in patients with major depressive disorder [182], and severe insomnia has been associated to lower BDNF [183]. Lastly, different inhibitors of TrkB were found to decrease REM sleep rebound after RSD [180]. Therefore, the literature suggests that the effects of Rhy on the BDNF/TrkB pathway could impact wakefulness and sleep phenotypes in numerous ways. However, the diverse roles of BDNF also suggest that the modulation by Rhy is likely context dependent.

### 2.5. ERK/MAPK Pathway

Rhy was shown to influence the phosphorylation (indicative of the activation) of ERK/MAPK. For instance, i.p. injection of Rhy diminished the elevated ERK phosphorylation (p-ERK) in trigeminal nucleus caudalis of rats stimulated with nitroglycerin (a rat migraine model) [82]. P-ERK level was also reported to be decreased by Rhy in rat and mouse microglia [71,76] and by *U. rhynchophylla* in murine macrophages [184]. In murine peripheral tissues, after several weeks of oral administration, Rhy was found to decrease the level of p-ERK in the lungs [100] and Isorhy to decrease it in the heart [101]. In contrast, others have reported that p-ERK levels were unaltered in the cortex or hippocampus after i.p. Rhy injections [86,87], which might be explained by a smaller dosage (i.e., 0.25 vs. 10–30 mg/kg). ERK and MAPK belong to a signaling cascade downstream of several membrane receptors, including NMDAR, TrkB, and toll-like receptors (TLRs), and can modulate multiple cellular responses via cAMP response element-binding protein (CREB) and activity-regulated genes such as *Arc*, *Dbp*, *Homer1a*, and *Bdnf* [163,164,165,166,185,186,187,188] (Figure 2). Therefore, the impact of Rhy on the ERK pathway may be linked to effects on both upstream and downstream elements.

CREB is a downstream effector of ERK/MAPK particularly relevant to understand the effects of Rhy. CREB is activated by neuronal activity and acts downstream of numerous other pathways including NMDAR and PI3K/AKT [164,166,187,188] (Figure 2). Rhy was shown to reduce p-CREB positive cells in the striatum and hippocampus in rats with meth and ketamine-dependent p-CREB increase [21,90,92]. Rhy was also found to rescue the meth-induced decrease in the number of c-fos positive cells in the striatum and CA1, which was suggested to depend on CREB [90].

With regard to the neurophysiology of sleep, the ERK pathway was shown to associate with both wake/sleep history and regulation. Indeed, ERK phosphorylation has been reported to increase after 15 min of wakefulness and to decrease after 15 min of NREM sleep in the mouse cerebral cortex [186]. Moreover, RSD was found to decrease p-ERK level in the rat hippocampus [189]. In parallel, the deletion of *Erk1* or *Erk2* genes, as well as the inhibition of ERK phosphorylation, was found to increase the time spent awake in mice, generally at the expense of NREM sleep [186]. The level of p-ERK was also reported to correlate with sleep time in *Drosophila* [190]. Interestingly, the inhibition of ERK phosphorylation was shown to increase NREM sleep delta power in mice [186]. In the cat visual cortex, ERK1 phosphorylation was observed to associate with REM sleep beta-gamma activity (20–40 Hz), and has been linked to REM sleep-dependent plasticity [191]. Several datasets are also supporting that sleep is regulated by CREB in both rodents and insects. For instance, mice mutant for CREB α and Δ isoforms show an increase in NREM sleep duration and a decrease in theta activity during wake and REM sleep [192]. Likewise, a specific mutation of CREB in forebrain excitatory neurons was found to reduce time spent awake and increase NREM sleep time and bout number in rats [193]. Moreover, SD was found to increase p-CREB in the rat cerebral cortex [194,195], but RSD decreases it in the rat hippocampus [189]. In flies, SD was found to enhance CREB transcriptional activity, while the inhibition of CREB activity was found to increase rest [196]. In sum, effects of Rhy on both ERK and CREB could impact wake/sleep duration and modulate EEG activity including NREM sleep delta power.

### 2.6. PI3K/AKT Signaling Network

The signaling by PI3K/AKT represents a major pathway regulating cell survival and growth [197]. Various receptors such as RTK and cytokine receptors directly stimulate PI3K upon ligand binding, which enables site-specific phosphorylation (and activation) of AKT by 3-Phosphoinositide-dependent protein kinase-1 (PDK1) and mechanistic target of rapamycin complex 2 (mTORC2) [198,199]. AKT controls numerous cellular processes such as apoptosis, anabolic metabolism, and angiogenesis notably via the phosphorylation of glycogen synthase kinase-3 (GSK3) and mTORC1 [200,201,202].

Both Rhy and Isorhy seem to activate the PI3K/AKT pathway [73,75,94,98,99] (Table 1). This pathway likely mediates neuroprotective effects of Rhy given that AKT induces anti-apoptotic and pro-survival effects [203,204,205,206]. In a Parkinson’s disease model in which cerebellar neurons are exposed to 1-Methyl-4-phenylpyridinium (MPP+, a potent neurotoxin), pre-treatment with Rhy was shown to decrease neuronal death [75]. This effect was abolished by the addition of a specific PI3K inhibitor, indicating that the effect of Rhy on cell survival is PI3K/AKT-dependent [75]. Also, Rhy and Isorhy were shown to prevent the shift towards apoptosis as measured with the Bax to Bcl-2 ratio [75,79,98]. In similar experimental conditions, *U. Rhyncophylla* has been shown to favor anti-apoptotic over pro-apoptotic proteins *in vitro* [207]. Moreover, Rhy, Isorhy and *U. Rhyncophylla* were all shown to prevent the increase of caspase-3 cleavage in various models of neurotoxicity [70,79,94,98,207,208]. The cleavage of caspase-3, known as an ‘executor of apoptosis’, is often considered the ultimate step in the apoptotic cascade [209].

GSK3, a major downstream effector of AKT [200], is a serine/threonine protein kinase particularly abundant in the CNS [210,211]. In mammals, GSK3 has two paralogs (i.e., homologous proteins derived from different genes), GSK3α and GSK3β [212]. Unlike most enzymes, GSK3 is constitutively active and pathways converging on it tend to decrease its activity by phosphorylation. GSK3 has repeatedly been linked to mood disorders [213,214]. The literature shows that Rhy inhibits GSK3β under pathological conditions, which mainly depends on the activation of PI3K/AKT. Indeed, Rhy was shown to reverse the decrease in GSK3β phosphorylation induced by MPP+ in cerebellar granule neurons, which was found to be PI3K-dependent [75]. Similarly, daily administration of Isorhy to chronically stressed mice or to Aβ-treated rats was reported to revert the decrease in GSK3β and AKT phosphorylation in the hippocampus and/or cerebral cortex [98,99]. Of interest is also that GSK3 is part of a pathway controlling NRF2 (nuclear factor E2 related factor 2) [215], which levels and translocation to the nucleus are enhanced by Rhy in hippocampal neurons of rats subjected to subarachnoid hemorrhage [208]. Isorhy had the same effect on NRF2 [74,101] and was also shown to induce transcription of ARE (antioxydant response element)-dependent genes [74]. The transcription of those genes is activated by NRF2 under oxidative stress conditions [216,217].

Few data are directly linking PI3K/AKT to sleep regulation. AKT was shown to respond to chronic sleep restriction, which decreases its phosphorylation in the hippocampus [218], thereby inhibiting the pathway. On the other hand, downstream targets of PI3K/AKT have been associated to sleep regulation, with in particular GSK3β activity that seems to impact sleep and the response to sleep loss. Firstly, mutant mice with constitutively active GSK3β were shown to have indications of an increased fragmentation of wakefulness and sleep states [219], and GSK3β knockdown in the cerebral cortex modifies the wakefulness and sleep EEG under baseline conditions and after SD in mice (Leduc et al. in preparation). Of note is that a genetic polymorphism decreasing GSK3β activity was found to ameliorate the clinical response to total SD in depressed patients [220,221]. Secondly, sleep-wake history appears to modify GSK3β activity. Chronic sleep restriction over a week was indeed shown to increase GSK3β phosphorylation in the hippocampus [218], and spontaneous wakefulness during the dark period to increase it in the hippocampus [222]. In a recent study, GSK3β activation was shown to occur at the transition to and during sleep and was proposed to act as major regulator of sleep-dependent plasticity [223]. In fact, GSK3β downregulation was found to abolish the SD-driven increase in mEPSCs (miniature excitatory post-synaptic currents) amplitude in the mouse PFC [224], supporting a role in wake/sleep-dependent plasticity. Thirdly, lithium, which is a direct inhibitor of GSK3 (α and β) [214], and the first-line treatment for bipolar disorders [225], was shown to affect sleep quality. For instance, lithium was reported to improve sleep efficiency in bipolar type I patients [226], to increase NREM sleep and decrease REM sleep in healthy volunteers [227], and to reduce REM sleep in mice [228]. The literature thus strongly supports a bidirectional relationship between GSK3 and sleep, which likely represents a key pathway by which Rhy could impact sleep architecture and EEG activity during sleep due to its inhibitory activity on GSK3β.

mTORC1, another serine/threonine kinase downstream of AKT [201,229], is an additional possible target of Rhy potentially underlying a role in wake/sleep regulation. Indeed, Rhy was shown to increase the phosphorylation of mTOR in a rat stroke model [94]. In parallel, sleep-wake history modifies mTORC1 activity, with sleep loss decreasing mTORC1 phosphorylation and thus attenuating mTORC1-dependent protein synthesis in the mouse hippocampus [230]. In addition, we have observed that mice heterozygous for mTOR are showing more SWA during wakefulness and REM sleep, and less theta activity during NREM sleep in comparison to wild-type mice (Areal et al., unpublished). Globally, considering that main downstream effectors of PI3K/AKT shown to be modulated by Rhy have been linked to sleep, these represent pathways by which Rhy could impact wake/sleep phenotypes.

### 2.7. NF-κB and Neuroinflammation

NF-κB is a transcription factor with implications in multiple cellular processes including neuroinflammation [231]. It can be activated by cytokine receptors and TLRs, which drive its nuclear translocation via the phosphorylation/degradation of NF-κB inhibitors (IkBs) [231]. The administration of Rhy has repeatedly been shown to diminish NF-κB activation in pathological contexts both *in vitro* [71,76,96,97] and *in vivo* [82,86,94,96,97] (Table 1). For example, in a rat nitroglycerin-induced migraine model, pre-treatment with Rhy almost completely prevented nuclear translocation of NF-κB in the trigeminal nucleus caudalis [82]. Moreover, it was shown that Rhy could decrease abnormal degradation of IkBα in pathological conditions such as treatments with lipopolysaccharide (LPS), nitroglycerin or 2,5-dimethoxy-4-iodoamphetamine [76,82,84,96,97]. In addition, there is growing literature supporting that Rhy reduces some effects associated with NF-κB activation: (i) the upregulation of pro-inflammatory cytokines such as interleukin-1β (IL-1β) and tumor necrosis factor α (TNFα) [71,76,84,85,96,97] and (ii) the increase in oxidative stress caused, in part, by nitric oxide (NO) [71,76,82,86]. Indeed, the incubation of rat microglial cells with LPS in the presence of Rhy for 24 h diminished the increase in NO, IL-1β and TNFα, and the increase in inducible NO synthase (iNOS) expression [71]. In contrast to its effect on iNOS-dependent NO synthesis, Rhy was shown to enhance endothelial NOS (eNOS)-dependent NO production in renal arteries of constitutively hypertensive rats via PI3K/AKT activation [73]. Thus, Rhy has different effects on NO synthesis depending on the context (here neuroinflammation/oxidative stress vs. vascular tone control). In pathological models such as ischemic brain injury and Tourette syndrome, Rhy was also shown to attenuate the upregulation of TLRs and MyD88 [94,97], the latter being an adaptor protein linking TLR activation to NF-κB nuclear translocation [232]. This led to the suggestion that the anti-inflammatory effects of Rhy in pathological contexts could result from an inhibition/downregulation of the TLR pathway [94,97]. However, a causative link remains to be defined. 

The effect of Rhy on NF-κB and related pathways could impact sleep, at least in pathological contexts. Indeed, Rhy reduces the pathological upregulation of IL-1β, TNFα and NO, which are proposed to act as somnogenic substances [233,234]. More precisely, the administration of IL-1β, TNFα and NO (or of their precursors) was shown to increase NREM sleep duration in different mammalian species [235,236,237,238,239,240]. Moreover, the inhibition of these molecules and/or their transcription factor NF-κB was shown to decrease NREM sleep duration, again in multiple mammals [235,236,237,239,241,242,243,244,245,246,247,248,249,250,251]. In addition, SD was shown to upregulate IL-1β, TNFα, NO, and even NF-κB [252,253,254,255], and the inhibition of IL-1β, TNFα and NO can also reduce/block the NREM sleep rebound that is normally caused by sleep loss [242,243,246,248,256]. Finally, the administration of both IL-1β and TNFα was shown to increase slow wave amplitude during NREM sleep [238,239,257], and the inhibition of IL-1β, TNFα and NOS (non-selective NOS inhibition) was shown to reduce NREM sleep SWA [243,247,249]. The reduced NREM sleep SWA was also observed after SD for the inhibition of IL-1β and TNFα [246,248]. Accordingly, Rhy administration could, by inhibiting/downregulating NF-κB and IL-1β, TNFα and NO, reduce NREM sleep amount and SWA in pathological contexts. However, given that Rhy was shown not to impact IL-1β, TNFα, and p-IkBα levels in peripheral tissues (e.g., cardiomyocytes and macrophages) of healthy mice [84] (Table 2), support for a modulatory role of Rhy on sleep via this pathway under normal physiological conditions remains to be collected. 

### 2.8. Neurotransmitters Signaling

Rhy has also been suggested to affect neurotransmitter signaling. For instance, a 3-min incubation with Rhy was shown to inhibit muscarinic acetylcholine receptor 1 (mAChR1) and serotonin receptor 2 (5-HT_2_)-mediated currents in xenopus oocytes [65]. Also, i.p. injection of Rhy in rats was found to decrease the release of 5-HT in the hypothalamus, and to increase it in the amygdala, cerebral cortex, and brainstem [61]. In this last study, dopamine (DA) release was increased in all these brain regions after Rhy administration [61]. Furthermore, Rhy was reported to rescue the amph-induced decrease of ACh, and the amph- and meth-induced increase in DA [88,91]. Rhy was also shown to attenuate the elevated DA and D2 receptor levels in the striatum of a rat Tourette syndrome model [96]. This provides support for a direct impact of Rhy on neurotransmitters in a manner that depends on the (patho)physiological condition and brain region (Table 1). Importantly, mAChRs and DA receptors are metabotropic receptors, which activity has respectively been linked to Kv1.2 channels and L-VGCCs [258,259] (Figure 2), emphasizing that Rhy could act at multiple levels of neurotransmitter function (see Section 2.1). 

Interestingly, ACh, 5-HT and DA are important wake/sleep modulators and components of the ascending arousal system [39]. Cholinergic activation in pontine regions increases cortical activation and REM sleep, and suppresses NREM sleep and SWA [44,260]. In fact, mAChR1 and mAChR3 seem important for REM sleep regulation in both rodent and healthy subjects [261,262]. Furthermore, mAChR1 and other mAChRs modulate thalamocortical and hippocampal oscillations [263,264,265,266,267,268,269]. This suggests that the inhibitory effect of Rhy on mAChR1 (or its modulation of ACh release) may decrease REM sleep and cortical activation and modify EEG activity.

5-HT, mainly originating from the RN, is another contributor to arousal [270], but its effects on wake/sleep regulation and EEG activity are more controversial. Indeed, optogenetic activation of dorsal RN 5-HT neurons was found to induce cortical activation and wakefulness [45,271], whereas the administration of 5-HT or drugs enhancing 5-HT transmission was shown to enhance EEG synchronization and sleep [270]. These opposite roles likely originate from the variety of 5-HT projections, such as to the BF [272], tegmental regions [273], and hypothalamic sleep regulatory neurons [274,275]. Moreover, different 5-HT receptors may be differentially involved [276], given that the activation of 5-HT_1A_ receptors can induce REM and theta activity [277,278,279,280], while that of 5-HT_1B_, 5-HT_2A_, 5-HT_2A/2C_ or 5-HT_7_ is suggested to reduce REM sleep [281,282,283,284,285]. Dopaminergic signaling was also found to be involved in wake/sleep regulation. Briefly, DA cells in the ventral tegmental area (VTA) discharge with different firing patterns during NREM and REM sleep [286], and DA stimulation in the VTA induces behavioral arousal [287]. Overall, more research is required to determine the mechanisms by which Rhy impacts 5-HT and DA neurotransmissions in order to eventually predict the 5-HT- and DA-dependent effects on sleep of Rhy.

Finally, the only literature directly linking Rhy and sleep (see also introduction) suggests that Rhy and Rhy-containing drugs are inducing sleep in rodents via GABA_A_ receptors. In fact, the sleep-promoting effects of the two *Uncaria*-containing drugs were found to be suppressed by the GABA_A_ receptor inhibitor bicuculline [57,58]. The only study using Rhy has linked the increased sleep time to increased level of GABA_A_ receptor subunits and increased glutamic acid decarboxylase (GAD)65/67 ratio (indicative of increased GABA synthesis at the synapse) in hypothalamic neurons [22]. Many GABAergic neurons regulate the activity of arousal and sleep circuits [39]. The majority of sedatives/hypnotics, such as benzodiazepines, are GABA_A_ receptor agonists and promote ‘light’ (as opposed to ‘deep’) NREM sleep [288,289]. In addition, GABAergic signaling is implicated in cell synchronization during sleep in brain circuits such as the thalamocortical network [30,34]. Therefore, GABAergic signaling is likely a pathway by which Rhy could increase sleep time and should be further investigated *in vivo*.

## 3. Conclusions

This review describes how Rhy affects diverse cellular pathways showing a particular relevance to sleep regulation, including VGCC, VGKC, NMDAR, RTK, ERK/MAPK, PI3K/AKT, NF-κB, and neurotransmitter signaling. The literature reveals both acute and delayed/chronic effects of Rhy on these different pathways. This suggests that Rhy may exert rapid effects on wakefulness/sleep quantity and quality, as well as effects that could last for some weeks after exposure. It is worth noting that the effects of Rhy on ion channels have only been characterized under acute conditions. This underlines the need to investigate the delayed and long-term effects of Rhy on ion channels in particular.

Interestingly, almost all studies describing effects of Rhy *in vivo* have reported effects solely under pathological/disturbed conditions (e.g., stress, treatments with psychostimulants, inflammation, animal models of diseases including stroke, epilepsy, and Alzheimer’s disease), and not in control animals. In fact, apart from effects of Rhy under normal/undisturbed conditions reported *in vitro* for ion channels, neurotransmitter receptors, NMDAR and BDNF, only two *in vivo* studies demonstrate effects of Rhy under normal conditions. In the first, Rhy altered DA and 5-HT levels in the rat hippocampus [61], whereas the second showed that Rhy increases total sleep time and REM sleep in rats [22]. Therefore, the literature suggests that Rhy impacts molecular/cellular pathways predominantly under disturbed/diseased conditions. This indicates that Rhy could be particularly beneficial for some pathological conditions involving sleep disturbances. Nevertheless, the physiological effects (assessed under normal conditions) of Rhy on molecular/cellular targets such as ERK/MAPK, NF-κB (and TLR), or D2 receptors should be characterized in the CNS, given that effects have only been described in the context of neurotoxicity, inflammation or epilepsy.

Sex-dependent effects of Rhy also represent an area of need for future research. Indeed, among all studies reviewed in this article, only three have studied females. Two of these used both sexes to show effects of Rhy on EphA4 phosphorylation or neurotransmitter levels [18,61] and did not report sex-dependent effects. The last study used only females and reported that Rhy reduces inflammatory responses and impacts the MAPK/ERK pathway in an asthma model [100], effects that are comparable to those in males reported in other studies [82,101]. Therefore, there is a clear need to investigate whether Rhy has sex-dependent effects. This is particularly relevant with regard to Rhy targets that have been shown to be differentially involved in sleep in the two sexes. For example, genetic variants in *CACNA1C* were associated with increased sleep latency in male infants but not in females [116].

Another neglected sleep-related research area concerns the potential for effects of Rhy on circadian functions. Many of the pathways presented in this review have been linked to the circadian timing system [290]. For instance, NMDARs (including the GluN2B subunit), TrkB receptors, and D2Rs show circadian rhythms of mRNA or protein levels in specific brain regions [291,292,293,294,295,296]. This strongly suggests that the effects of Rhy on these specific targets will depend on time-of-day and/or internal circadian time. Thus, it appears crucial to consider the effects of Rhy separately, for instance, for the light and dark periods, at least for targets with known circadian regulation. Such investigation would notably help to determine the relevance of Rhy in chronotherapy.

This review has compiled the effects of Rhy with a particular focus on the CNS. However, Rhy impacts, among others, the cardiovascular and immune systems [3,10,84,297] (see also Section 2.1 and Section 2.7). Rhy was indeed shown to have antihypertensive roles via anti-sympathetic and vasodilatory effects that are mainly linked to ion channels [10]. Heart rate and heart rate variability differ between sleep stages [298,299], while systemic inflammation impacts sleep [28]. Thus, future research on Rhy should also consider the interplay between peripheral tissues and sleep.

As indicated in the introduction, Rhy is one of the most abundant alkaloids in *Uncaria*, which has been highly used in Chinese and Japanese traditional medicine [3,4,10]. The composition of *Uncaria* and, as a consequence, the components present in traditional treatments such as Chotoko could vary depending on the geographic region and plant growing conditions [300]. This may explain variations in the therapeutic effects of *Uncaria*, which might be overcome by the use of purified Rhy. Therefore, describing the specific mechanisms of action of Rhy will help defining the medical applications of this chemical. Nevertheless, multiple compounds in *Uncaria* may have synergistic actions in contributing to health benefits associated with the plant (e.g., chemicals helping the absorption of others [301]). Thus, studies comparing the benefits of Rhy to those of blends of *Uncaria* will help to identify the best treatment strategies for sleep disturbances and associated pathological conditions.

To conclude, Rhy may impact sleep architecture and oscillations by targeting a diversity of cellular pathways. These effects may specifically underlie the impacts of Chotoko, YKS, and other *Uncaria* treatments on sleep. Further studies are required to precisely determine the effects of Rhy on sleep as well as on other CNS functions (e.g., memory) under undisturbed/normal conditions. A better understanding of the cellular mechanisms of action of Rhy that are relevant to sleep physiology may eventually help to determine whether this alkaloid could be used in sleep medicine. 

## Figures and Tables

**Table 1:** Compilation of datasets showing molecular and cellular (and some electrophysiological and behavioral) effects of rhynchophylline (Rhy) organized as a function of treatment type and duration, and by measurement timing.

Rhy Application	Timing of Measurement	Rhy Effect	Model	Reference
**INCUBATIONS**				
20 s	Immediate	Attenuates epilepsy-induced ↑ in NMDAR current in EC slices	Rat brain slices	[19]
80 s	Immediate	Accelerates activation and inactivation of VGKC Accelerates activation and inactivation of Kv1.2	N2A cells HEK293	[64]
3–8 min	Immediate	↓ mAChR1- and 5-HT_2_-mediated currents (effect disappears after 1 min)	Xenopus oocytes	[65]
		Attenuates epilepsy-induced ↑ of EC neuron discharge frequency	Rats	[19]
		↓ open time and ↑ close time of L-VGCCs	Rat cortical neurons	[66]
		↓ Ca^2+^ influx via L-VGCCs	Rat cardiomyocytes	[67]
		Non-competitive inhibition of NMDAR current	Xenopus oocytes	[68]
15–30 min	Immediate	↓ EfnA1-dependent EphA4 phosphorylation and EphA4 clusters	Rat cortical neurons	[18]
		Attenuates ischemia-induced ↓ in population spike amplitude	Rat hipp. slices	[65]
		↓ Ca^2+^ intracellular increase via L-VGCC, promotes vasodilation	Human artery smooth muscle cells	[69]
1 h	Immediate	Attenuates ischemia-induced ↑ in ROS, MDA, LDH, mPTP, AIF, Ca^2+^ and caspase 3 and 9 mRNA and protein Attenuates ischemia-induced ↓ in mitochondrial membrane potential, SOD, GPx, Cytc	Rat cardiomyocytes	[70]
		↑ GAD65/67 and GABA_A_R subunits expression	Rat hypothalamic neurons	[22]
30 min	2 h post Rhy	Attenuates Aβ-induced ↑ in EphA4 phosphorylation and LTP impairment	Rat hipp. slices	[18]
2–6 h	Immediate	Attenuates LPS-induced ↑ in *Cox2*, *iNos*, *Ccl2* mRNAs	Rat microglia	[71]
		↑ *Grin1* mRNA (no difference in *Grin2b*)	Rat hipp. neurons	[72]
12 h	Immediate	Improves endothelial relaxation and ↑ p-Src, p-AKT and NO (in hypertensive rat arteries) and ↑ p-eNOS (in WT arteries)	Rat intrarenal arteries	[73]
24 h	Immediate	Attenuates LPS-induced ↑ in p-ERK, p-38, p-IkBα, NFκBp65 Attenuates LPS-induced ↓ in IkBα Attenuates LPS-induced ↑ in culture medium MCP1, PGE2, NO, IL1β, TNFα	Rat microglia	[71]
1 h	24 h post Isorhy	* Attenuates MPP-induced ↑ in p-GSK3β Tyr297, p-FYN and ROS * ↑ nuclear NRF2 and ARE transcriptional activity	Human SH-SY5Y neuroblastoma cells	[74]
2 h	24 h post Rhy	Attenuates MPP-induced ↓ in p-GSK3β Ser9, p-AKT and MEF2D Attenuates MPP-induced ↑ in Bax/Bcl-2 ratio	Rat granule neurons	[75]
48 h	Immediate	↑ *Grin1* mRNA and GluN1, and ↓ *Grin2b* mRNA and GluN2B	Rat hipp. neurons	[72]
		Attenuates LPS-induced ↑ in NO, iNOS, TNFα, IL-1β, p-p38, p-ERK Attenuates LPS-induced ↓ in IkBα	N9 mouse microglia	[76]
		↓ GluN1 and ↓ ketamine-induced ↑ in GluA2/3	PC12 cells	[77]
72 h	Immediate	↑ proliferation, GluN1, GluN2B, GluN3A ↓ BDNF, OXTR, and ATP Alters proliferation/differentiation related genes	Bone mesenchymal human cells	[78]
24 h	48 h post Rhy	Attenuates MPP-induced ↑ ROS, LDH, Caspase-3 activity and apoptosis; Attenuates MPP-induced ↓ Bcl2/Bax ratio and p-AKT	PC12 cells	[79]
**SINGLE ADMINISTRATIONS**				
IC	100–600 s post Rhy	Attenuates Aβ-induced ↑ in the frequency of spontaneous discharge in CA1	Rats	[80]
IV	30 min post Rhy	Attenuates ischemia-induced ↓ in 5HIAA and DOPAC in striatum and hipp. Attenuates ischemia-induced ↑ of NE in striatum and hipp.	Rats	[81]
IP	50 min post Rhy	↓ DA in cortex, hypothalamus, and brainstem ↓ 5-HT in amygdala ↑ 5-HT in hypothalamus, and ↓ 5-HT release in hypothalamic slices ↑ 5-HT release in cortex, amygdala, and brainstem slices ↑ DA release in cortex, hypothalamus, amygdala, and brainstem slices ↓ righting reflex and spontaneous locomotor activity	Rats	[61]
Oral	0–6 h post Rhy	↓ locomotor activity and sleep latency, ↑ total sleep time ↓ number of sleep/wake cycles, ↑ total sleep time and REM sleep	Mice and Rats	[22]
IP	48 h post Rhy	Attenuates stress-induced ↑ p-EphA4, p-FYN, p-Cdk5, p-Ephexin in PFC, CA3, DG Attenuates stress-induced ↓ BDNF, p-TrkB, PSD95, spines in PFC, CA3, DG	Mice	[17]
IP	52 h post Rhy	Attenuates NTG-induced ↑ in EEG theta and delta activity, oxidative stress (GSH, blood CGRP), p-ERK1/2, p-JNK, p-p38, p-IκBα, and nuclear NF-κB p65 (all in trigeminal nucleus caudalis)	Rats	[82]
Hipp. inj	2 w post Rhy	Attenuates Aβ-induced ↑ cell death, GluN2B, and NMDA Ca^2+^ influx in CA1	Rats	[83]
**MULTIPLE ADMINISTRATIONS**				
SC for 3 days	1–3 h after last injection	Attenuates LPS-induced ↓ in stroke volume and cardiac output Attenuates LPS-induced ↑ in IL-1β, TNFα and p-IkB*α* in heart, macrophages and serum	Mice	[84]
IP for 3 days	3 h after last injection	* Attenuates KA-induced epileptic seizures ** Alters levels of *Bdnf*, *Fos*, *Nfkbia*, *Map2k3*, *Il1b* in cerebral cortex and hipp.	Rats	[85]
		Attenuates KA-induced epileptic seizures		[86]
		Attenuates KA-induced epileptic seizures and KA- induced ↑ in hippocampal p-JNK ** Attenuates KA-induced ↓ in cortical IL-6		[87]
IP for 3 days	12 h after last injection	Attenuates meth-induced ↑ in 5-HT, DA, TH, Glut, GluN2B, and locomotion	Zebrafishes	[88]
		Attenuates meth-induced ↑ in GluA1 and CPP		[89]
		Attenuates meth-induced ↑ in p-CREB and c-fos positive cells in CA1 and striatum	Rats	[90]
		Attenuates amph-induced ↑ in CPP, glutamic acid, DA, and NE Attenuates amph-induced ↓ in GABA, endorphin, and ACh		[91]
		Attenuates ketamine-induced ↑ in CPP, *Nr4a2* and *Bdnf* mRNAs, NURR1, BDNF, p-CREB (all hipp.)		[21,92]
		Attenuates amph-induced ↑ in CPP and *Grin2b* mRNA, and GluN2B protein in mPFC and CA1		[20]
		Attenuates meth-induced ↑ in CPP and GluN2B in brain tissue	Mice	[93]
IP for 3 days	24 h after last injection	Attenuates KA-induced ↑ in IL-1β and BDNF positive cells in cortex and hipp.	Rats	[85]
		Attenuates KA-induced ↑ NO scavenging activity in blood		[86]
IP for 5 days	24 h after last injection	↓ brain infarction and neurological deficits in a stroke model In cerebral cortex: Accentuates ischemia-induced ↑ in p-AKT and p-mTOR Attenuates ischemia-induced ↑ in TLR2,4, MyD88, caspase 3, and nuclear NF-κB Attenuates stroke-induced ↓ in p-BAD, BDNF, *Bdnf* and claudin-5		[94]
ICV infusion for 9 days	33–34 h after ICV	Attenuates epilepsy-induced ↑ EC discharge frequency, neuronal death and GluN2B and Nav1.6	Rats	[19]
1 week gavage	1 week after last gavage	Attenuates cytotoxicity-induced ↓ in TH-positive cells in substantia nigra	Mice	[79]
2 weeks gavage	Immediate	** Attenuates KA-induced neuronal death and KA-induced ↑ in spike amplitude	Rats hipp. slices	[95]
3 weeks oral	Not specified	Attenuates DOI-induced ↑ TNFα, IL-6, and IL-1B (in serum and striatum); Attenuates DOI-induced ↑ p-NF-κB p65, p-IkB*α*, TLR2, caspase1, MyD88, DA, D2R (in striatum) Attenuates DOI-induced ↓ in p-TrkB, BDNF (in striatum), and cell viability	Rats	[96,97]
3 weeks gavage	24 h after last gavage	* Attenuates Aβ-induced ↓ in p-AKT, p-GSK3β (in brain), Bcl2/Bax in hipp., and memory * Attenuates Aβ-induced ↑ in caspases 3 and 9 in hipp.	Rats	[98]
3–4 weeks gavage	Immediate	Attenuates p-EphA4 and rescues LTP in hipp. slices in APP mice	Mice	[18]
3 weeks gavage	5 days after last gavage	* Attenuates chronic mild stress-induced ↓ p-AKT, p-GSK3β, BDNF, NGF in cortex and hipp., and sucrose preference * Attenuates chronic mild stress-induced ↑ in TNFα, IL-6, nuclear NF-κB in cortex and hipp., and locomotion	Mice	[99]
1 day gavage/week for 4 weeks	24 h after last gavage	Attenuates asthma-induced ↑ in eosinophil recruitment, IL-13, IL-4, IL-5 in serum Attenuates asthma-induced ↑ TGFβ, Smad4, p-Smad2, p-Smad3, p-ERK1/2 and p-38 in lung tissue	Mice	[100]
6 weeks in food	Immediate	* Attenuates cardiac hypertrophy-induced ↑ in TGFβ1, cTGF, Collagen_1,3_, p-ERK, p-38, p-JNK, and attenuates the induced ↓ in SOD2 * ↑ NRF2 and accentuates the induced ↑ in SOD3	Mice	[101]

* Studies with Isorhynchophylline. ** Studies with *Uncaria rhynchophylla*. Upward arrows are indicating an increase and downward arrows a decrease; 5-HT: 5-hydroxytryptamine or serotonin; 5HIAA: 5-hydroxyindoleacetic acid; 5-HT_2_R: serotonin receptor 2; Aβ: amyloid β; AIF: apoptosis-inducing factor; ACh: acetylcholine; AKT: RAC serine/threonine-protein kinase; amph: amphetamine; APP: amyloid precursor protein; ARE: antioxidant response element; ATP: adenosine triphosphate; BAD: Bcl-2-associated death protein; BDNF: brain-derived neurotrophic factor; CA1: hippocampal cornu ammonis-1; CA3: hippocampal cornu ammonis-3; Cdk5: cyclin dependent kinase 5; CGRP: calcitonin gene-related peptide; Ccl2: monocyte chemoattractant protein 1 gene; Cox2: cyclooxygenase 2; CPP: conditioned place preference; CREB: cAMP response element-binding protein; cTGF: connective tissue growth factor; Cytc: cytochrome c; D2R: dopamine D2 receptor; DA: Dopamine; DG: dentate gyrus; DOI: 1-(2,5-dimethoxy-4-iodophenyl)-2-aminopropane; DOPAC: 3,4-Dihydroxyphenylacetic acid; EC: entorhinal cortex; EEG: electroencephalographic; eNOS: endothelial nitric oxide synthase; EfnA1: ephrin A1; EphA4: Eph receptor A4; ERK: extracellular signal-regulated kinases; FYN: tyrosine-protein kinase Fyn; GABA_A_R: gamma-aminobutyric acid type A receptor; GAD: glutamic acid decarboxylase; GluA: α-amino-3-hydroxy-5-methyl-4-isoxazolepropionic acid (AMPA) receptor subunit; GluN: NMDAR subunit; GPx: glutathione peroxidase; Grin2b: glutamate ionotropic receptor NMDA type subunit 2B; GSH: glutathione; GSK3β: glycogen synthase kinase-3 β; Hipp.: hippocampus; IC: intracerebral; ICV: intracerebroventricular; IκBα: NF-kappa-B inhibitor alpha; iNOS: inducible nitric oxide synthase; IL: interleukin; IP: intraperitoneal; IV: intravenous; JNK: c-Jun N-terminal kinase; KA: kainic acid; Kv: VGKCs subunit; LPS: lipopolysaccharide; LTP: long term potentiation; L-VGCC: L-type voltage-gated calcium channel; MCP1: monocyte chemoattractant protein 1; MDA: malondialdehyde; MEF2D: myocyte enhancer factor 2D; meth: methamphetamine; mPFC: medial prefrontal cortex; MPP: 1-methyl-4-phenylpyridinium; mPTP: mitochondrial permeability transition pore; mTOR: mechanistic target of rapamycin; MyD88: myeloid differentiation primary response protein; NAc: nucleus accumbens; Nav1.6: voltage-gated sodium channel 1.6; NE: norepinephrine; NF-κB: nuclear factor-kappa B; Nfkbia: IκBα gene; NGF: nerve growth factor; NMDAR: N-methyl-D-aspartate receptor; NO: nitric oxide; Nr4a2: nuclear receptor subfamily 4 group A member 2 gene; NRF2: nuclear factor E2 related factor 2; NTG: nitroglycerin; Nurr1: nuclear receptor related-1 protein or nuclear receptor subfamily 4 group A member 2; OXTR: oxytocin receptor; PC12: cell derived from phaeochromocytoma of rat adrenal medulla; PSD95: postsynaptic density protein 95; REM: rapid eye movement; SC: subcutaneous; Smad: homolog of *Drosophila* mothers against decapentaplegic; SOD: superoxide dismutase; Src: proto-oncogene tyrosine-protein kinase Src; TGFβ: transforming growth factor beta; TH: tyrosine hydroxylase; TLR: toll-like receptor; TNFα: tumor necrosis factor α; TrkB: tropomyosin or tyrosine receptor kinase B; VGKC: voltage-gated potassium channel.

**Table 2:** List of literature showing effects of Rhynchophylline (Rhy) on sleep-related pathways under physiological (baseline) and/or pathological (disease-modeled) conditions.

	Effects under Baseline and/or Pathological Conditions	Sex(es) Studied	Reference
**VGCC**	**Baseline conditions**	Males	[67,103]
	**Baseline conditions**	**Males and females**	[104]
	**Baseline conditions**	Not indicated	[69]
	Pathological conditions	Not indicated	[66]
**VGKC**	**Baseline conditions**	Male and female cell lines	[64]
**NMDAR**	**Baseline conditions**	Not indicated	[68,72,78]
	Pathological conditions	Males	[19,88]
	Pathological conditions; no effect under baseline	Males	[20,83]
	Pathological conditions	Not indicated	[93]
**EPHA4**	Pathological conditions; no effect under baseline	**Males and females**	[18]
	Pathological conditions; no effect under baseline	Males	[17]
**BDNF/TRKB**	**Baseline conditions**	Not indicated	[78]
	Pathological conditions	Males	[85,92,94]
	Pathological conditions; no effect under baseline	Males	[17,99] *
	Pathological conditions	Not indicated	[21]
**ERK/MAPK**	Pathological conditions	Male cell line	[76]
	Pathological conditions	Not indicated	[71]
	Pathological conditions	Female	[100]
	Pathological conditions	Males	[82,86,87]
	Pathological conditions; no effect under baseline	Males	[101] *
**CREB**	Pathological conditions	Males	[92]
	Pathological conditions	Not indicated	[21,90]
**PI3K/AKT**	Pathological conditions	Males	[73]
	Pathological conditions	Male cell line	[79]
	Pathological conditions	Not indicated	[70]
	Pathological conditions; no effect under baseline	Not indicated	[75]
	Pathological conditions; **only one effect under baseline**	Males	[101] *
	Pathological conditions	Males	[94]
	Pathological conditions; no effect under baseline	Males	[98,99] *
**NF-κB**	Pathological conditions	Male cell line	[76]
	Pathological conditions	Not indicated	[71]
	Pathological conditions	Males	[82,85,86,94,97]
	Pathological conditions; no effect under baseline	Males	[84]
**Other NTs**	**Baseline conditions**	Not indicated	[65]
	**Baseline conditions**	**Males and females**	[61]
	Pathological conditions	Males	[88,96]
	Pathological conditions; no effect under baseline	Not indicated	[91]
**GABA_A_R**	**Baseline conditions**	Male neurons	[22]

* Studies with Isorhynchophylline. Lines with grey background denote *in vitro* measurements only. Studies showing Rhy effects under baseline conditions and/or including both sexes are in bold. Studies have not tested the effect of Rhy under baseline conditions if it is not indicated. AKT: RAC serine/threonine-protein kinase; BDNF: brain-derived neurotrophic factor; CREB: cAMP response element-binding protein; EphA4: Eph receptor A4; ERK: extracellular signal-regulated kinases; GABA_A_R: gamma-aminobutyric acid type A receptor; VGCC: voltage-gated calcium channels; NF-κB: nuclear factor-kappa B; NMDAR: *N*-methyl-d-aspartate receptor; NTs: neurotransmitters; PI3K: phosphoinositide 3-kinase; TrkB: tropomyosin or tyrosine receptor kinase B; VGKC: voltage-gated potassium channels.

## Data Availability

Not applicable.
